# Depression and anxiety disorders in a sample of facial trauma: A study from Iran

**DOI:** 10.4317/medoral.21068

**Published:** 2016-03-06

**Authors:** Sayed-Amir-Hossein Gandjalikhan-Nassab, Sahand Samieirad, Mohammad Vakil-Zadeh, Raha Habib-Aghahi, Maryam Alsadat-Hashemipour

**Affiliations:** 1 DDS. Member of Kerman dental and oral diseases research center, Kerman University of Medical Sciences, Kerman, Iran; 2 DDS, MSc. Assistant Professor of Oral and maxillofacial surgery, Department of Oral and Maxillofacial Surgery, School of; 3 Dentistry, Mashhad University of Medical Sciences, Mashhad, Iran 2DDS. Dentist, Dental School, Kerman University of Medical Science, Kerman, Iran; 4 DDS, MSc. Member of Kerman dental and oral diseases research center, Kerman University of Medical Sciences, Kerman, Iran; 5 DDS, MSc. Member of Kerman Social Determinants on Oral Health Research Center, Kerman University of Medical Sciences, Kerman, Iran and Kerman Dental and Oral Diseases Research Center, Kerman University of Medical Sciences, Kerman, Iran. Associate Professor of Oral Medicine, Department of Oral Medicine, Dental School, Kerman University of Medical Science, Kerman, Iran

## Abstract

**Background:**

Various studies have shown that such patients are susceptible to psychological problems and poor quality of life. The aim of the present study was to evaluate and compare the prevalence of depression and anxiety disorders and quality of life in a group of facial trauma.

**Material and Methods:**

In the present cross-sectional study Hospital Anxiety and Depression Scale (HADS) and Oral Health Impact (OHIP-14) questionnaires were used. In this study, fifty subjects were selected from the patients with maxillofacial traumas based on the judgment of the physicians, referring to hospitals in Kerman and Rafsanjan during 2012-2013. In addition, 50 patients referring to the Dental School for tooth extraction, with no maxillofacial traumas, were included. SPSS 13.5 was used for statistical analysis with two-sample t-test, Mantel-Haenszel technique, Pearson’s correlation coefficient and chi-squared test.

**Results:**

Seven patients with maxillofacial traumas were depressed based on HADS depression scale, with 5 other borderline cases. However, patients referring for surgery or tooth extraction only 2 were depressed and 1 patient was a borderline case. The results showed that patients with maxillofacial traumas had higher rates of depression and anxiety, with significant differences between this group and the other group (*P*=0.01). The results of the present study showed a significant prelateship between depression severity and confounding factors. The mean of OHIP-14 parameters were 35.51 ±5.2 and 22.3±2.4 in facial trauma and dental surgery groups, respectively, with statistically significant differences (*P*=0.01).

**Conclusions:**

The results of the present study showed depression and anxiety disorders in patients with maxillofacial trauma. The results showed a higher rate of anxiety and anxiety in patients with maxillofacial traumas compared to the control group.

**Key words:**Depression, facial trauma, HADS, OHIP-14, questionnaire.

## Introduction

Maxillofacial traumas are one of the general health problems in both developing and developed countries. The majority of these injuries result in long-term disabilities and deformities, with social and psychological outcomes. Various studies have shown that such patients are susceptible to psychological problems and poor quality of life. In addition, they face the problem of short- and long-term psychological problems (1-3).

The serious social‒psychological outcomes of facial injuries have attracted enormous attention in recent years; however, there is insufficient comparative data between different populations in relation to the prevalence of psychological anxietyes subsequent to these injuries (4-7,1). Studies have shown that there are significant similarities in the epidemiology of maxillofacial injuries all over the world. These injuries particularly affect young men and the most common etiologic factors are traumas due to fights, road accidents, sports injuries and industrial accidents (2,8,9).

Management of treatment and surgery of maxillofacial injuries is a challenging area. Traumas due to disturbances in the specific functions in the maxillofacial area might give rise to psychological injuries in these patients. In addition, these traumas might result in facial deformities or pave the way for chronic diseases. These injuries might influence the patients’ self-confidence and lead to various long-term social-psychological problems (1,4,5,7).

The incidence of psychological problems after facial traumas depends on the criteria used in different studies. Approximately 20-30% of adult patients with facial traumas exhibit symptoms and signs of anxiety. Both anxiety and depression are considered psychological disorders. While various treatments are available for such problems, unfortunately these conditions in patients with maxillofacial injuries are not diagnosed and usually remain untreated (10). Studies have shown that patients with maxillofacial injuries suffer from severe anxiety disorders. Researchers have concluded that management of preoperative anxiety is still a major challenge in maxillofacial surgery and knowledge about these problems and finding ways to decrease psychological anxiety are very important for both patients and surgeons (1-3,6). Despite increased global awareness of these problems, there is still inadequate comparative data on psychological anxiety in maxillofacial traumas. The aim of the present study was to evaluate and compare the prevalence of depression and anxiety disorders and quality of life in a group of facial trauma. This study is first study in Iran about depression and anxiety disorders and quality of life in a group of facial trauma.

## Material and Methods

In this study, all patients were completed Informed consent form before including in the study. In the present cross-sectional study Hospital Anxiety and Depression Scale (HADS) and Oral Health Impact (OHIP-14) questionnaires were used. The OHIP-14 questionnaire consists of 14 questions. In all these questions the interviewee is asked to answer each question in relation to the problems with his/her teeth, the oral cavity or dentures based on his/her experiences during the past 12 months. The answers are recorded based on Likert scale as follows: score 4: almost always; score 3: in most cases; score 2: sometimes; score 1: seldom; and score 0: never. Therefore, the score range will be 0-56. Higher scores indicate poor oral health-related quality of life. The Persian version of the questionnaire has been validated by Navabi *et al.* (11).

HADS questionnaire is used to evaluate anxiety and anxiety of patients. It was designed by Zigmond and Snaith (12) in 1983 and is used to determine the anxiety and depression severities a patient experiences. It is the most suitable tool to determine the psychological anxiety of patients with physical problems and is under the least influence of other physical ailments. The questionnaire consists of 14 questions, 7 of which relate to anxiety and 7 relate to depression. Each question might receive a score of 0-3 (0=never, 1=seldom, 2=sometimes & 3= always), indicating that each individual’s score on anxiety or depression might have a range of 0-21. Scores of 11 or more on the anxiety and depression subscales of this scale indicate anxiety and depression, scores of 8-10 represent a borderline score and scores of 0-7 represent a no depression/anxiety score. The questionnaire was confirmed in relation to its validity and reliability by Montazeri *et al.* (13)

In the next stage, the questionnaire was given to patients and explanations were provided in relation to the aim(s) of the study. Only those patients willing to take part in the study were given the questionnaire and were included in the study. The subjects were assured that all the data on the questionnaires will be kept confidential and will only be evaluated and analyzed from a statistical point of view. All the questionnaires were anonymous.

In this study, fifty subjects were selected from the patients with maxillofacial traumas based on the judgment of the physicians, referring to hospitals in Kerman and Rafsanjan during 2012-2013. In addition, 50 patients referring to the Dental School for surgery tooth, with no maxillofacial traumas, were included. The demographic data of the patients were also recorded, including age, gender, occupation, educational level and marital status. Furthermore, a history of psychological problems (based on consult with a psychiatrist) and the medications taken were recorded.

- Ethical considerations

Ethical considerations were taken into account throughout the study, and the patients’ names and medical information remained completely confidential. The subjects’ medical history was used solely for the purposes of the current study. The research proposal was approved by the ethics committee of Kerman University of Medical Sciences with the 542.93.k code.

- Statistical analysis 

SPSS 13.5 was used for statistical analysis with two-sample t-test, Mantel-Haenszel technique, Pearson’s correlation coefficient and chi-squared test.

## Results

A total of 100 patients were evaluated in the present study.  Table 1 presents the demographic data of the subjects.  Table 2 presents the results of comparisons made in relation to depression and anxiety between the two groups. Seven patients with maxillofacial traumas were depressed based on HADS depression scale, with 5 other borderline cases. However, patients referring for surgery or tooth extraction only 2 were depressed and 1 patient was a borderline case. The results showed that patients with maxillofacial traumas had higher rates of depression and anxiety, with significant differences between this group and the other group (*P*=0.01). In addition, the mean HADS score in the maxillofacial trauma group was higher than that in other group ( Table 3). There were significant differences in depression rates between the maxillofacial trauma patients and the patients in the other group. The odds ratio (OR) for depression in patients with maxillofacial traumas was calculated at 8.12 (OR=0.12, 95% CI: 3.65-30.5, *P*=0.001). The odds ratio for anxiety in maxillofacial trauma patients was calculated at 2.55 (OR=2.55, 95% CI: 0.90-7.15, *P*=0.07). The odds ratio in subjects referring for surgery or tooth extraction was calculated at 1.7 (OR=1.7, 95% CI: 0.5-4.12, *P*=1.1).  Table 4 shows the relationship between maxillofacial trauma, anxiety and depression scores and the confounding factors. The results of the present study showed a significant prelateship between depression severity and confounding factors. In relation to anxiety, a significant relationship was detected only with gender.

**Table 1:**
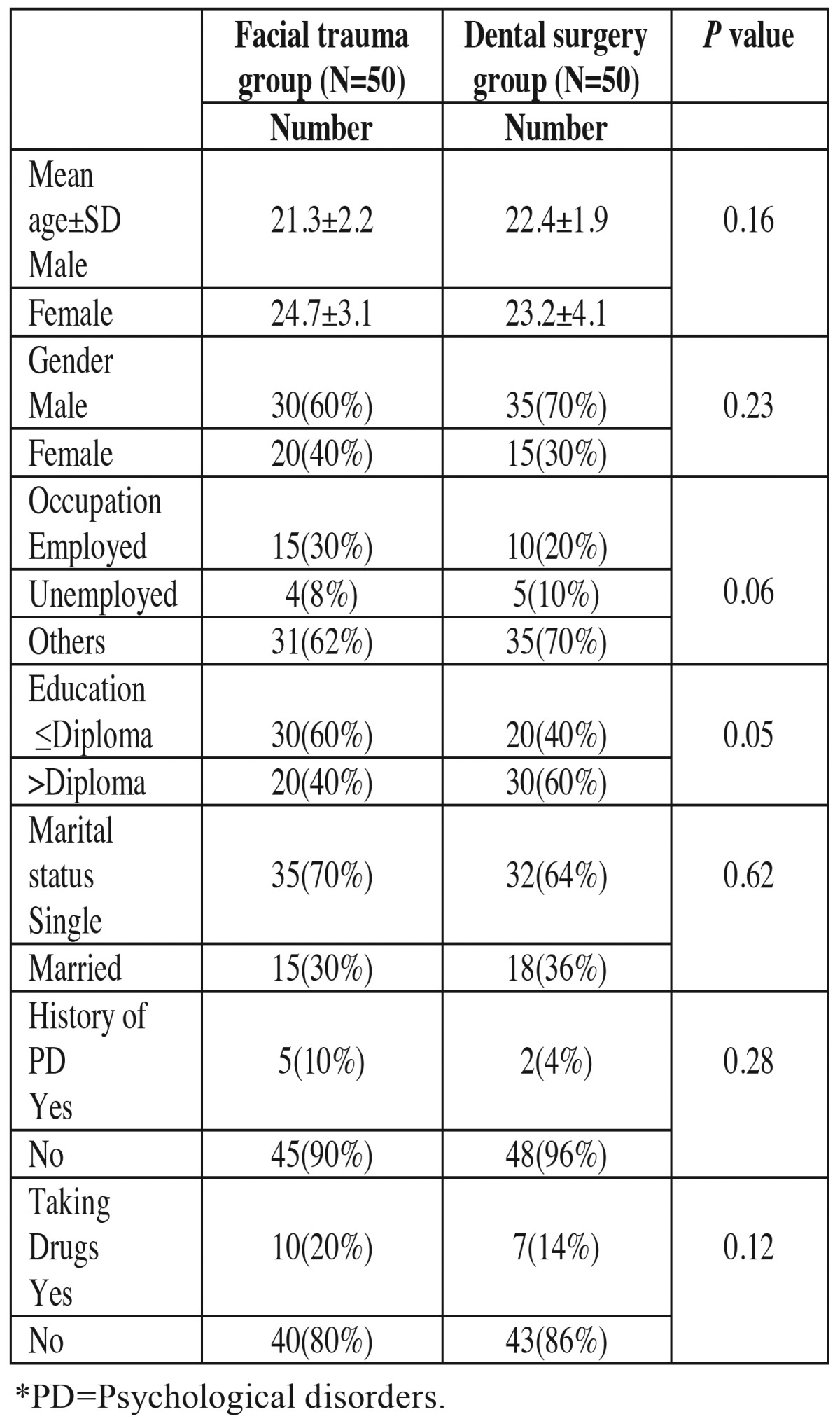
Baseline characteristics of the study sample.

**Table 2:**
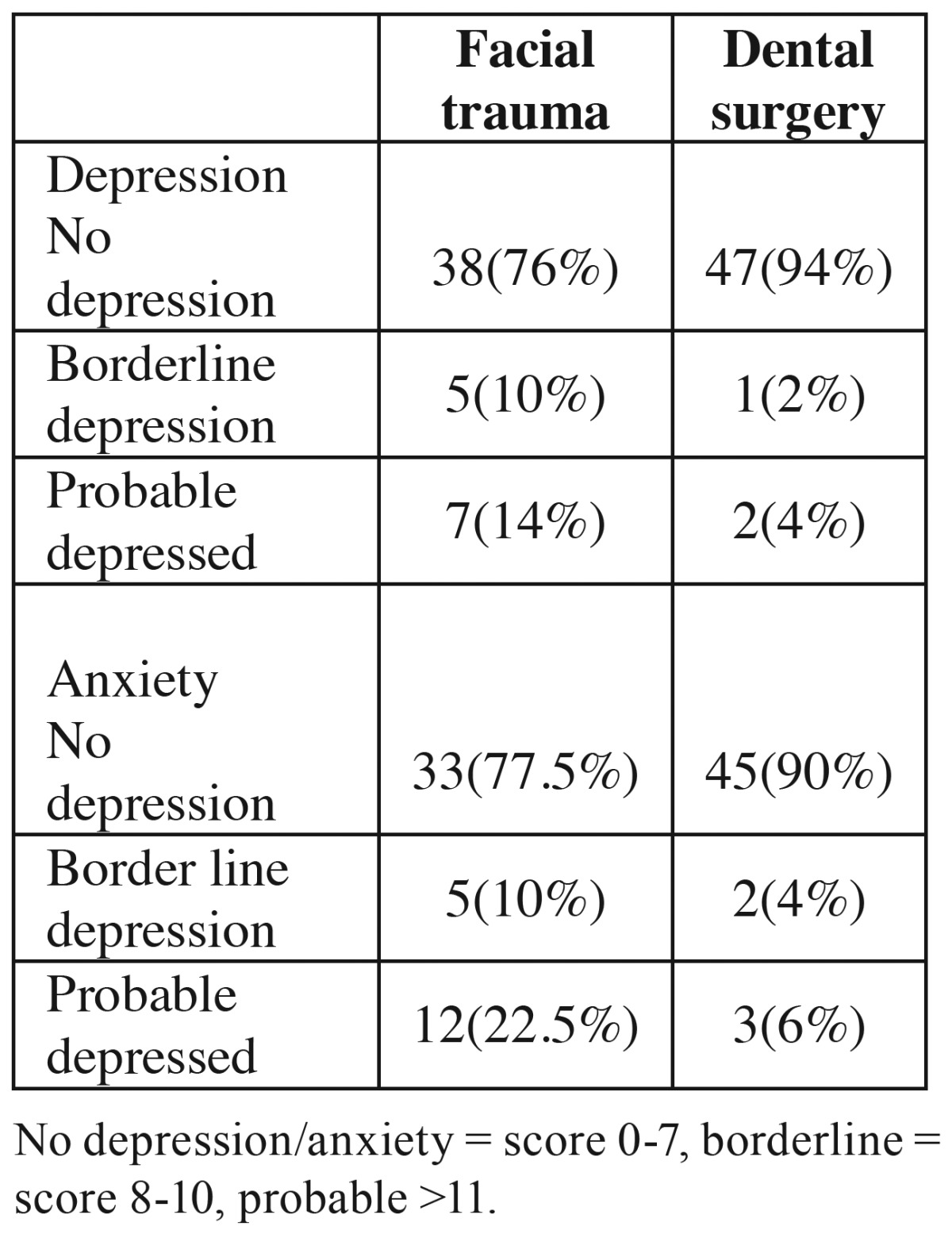
Analyses of data for both HADS-depression and anxiety subscales.

**Table 3:**
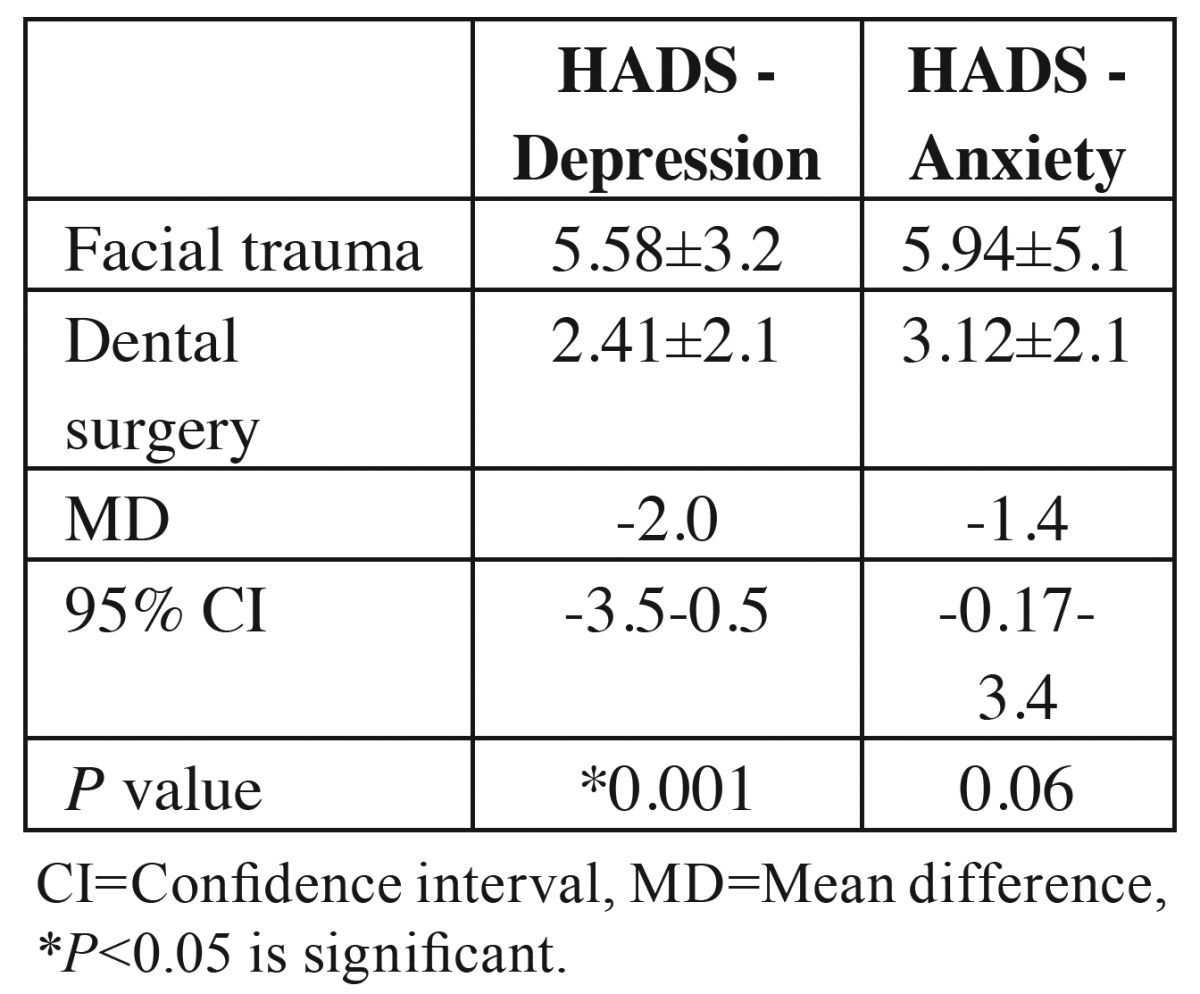
Comparison of Hospital Anxiety and Depression Scale scores in both facial trauma and control group.

**Table 4:**
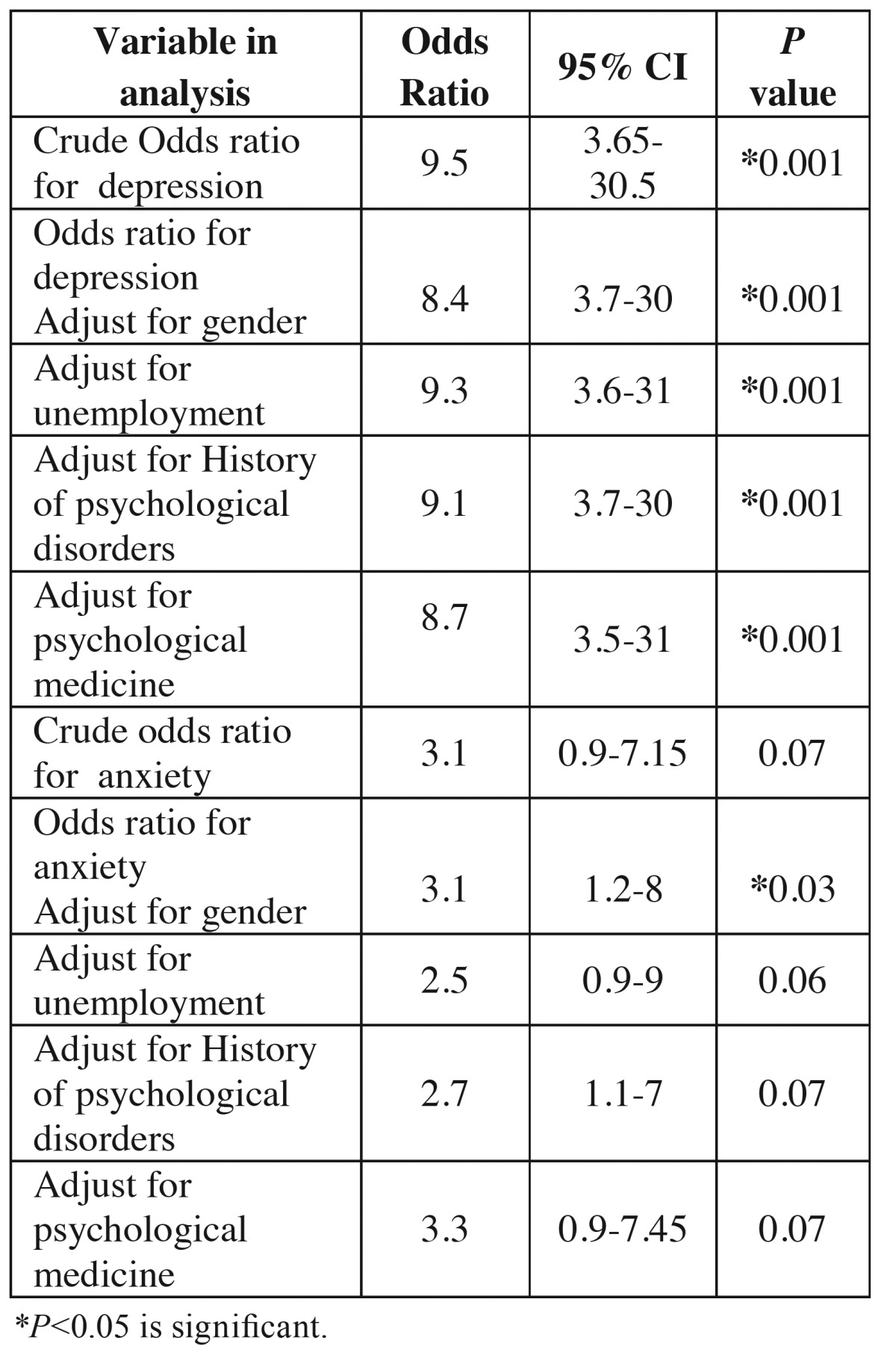
The association between facial trauma and depression and anxiety disorder. Odds ratios and 95% confidence intervals are given for crude and Mantel-Haenszel adjusted ratios.

The results showed a lower quality of life in patients with maxillofacial trauma, with a significant relationship in this respect. The mean of OHIP-14 parameters were 35.51 ±5.2 and 22.3±2.4 in facial trauma and dental surgery groups, respectively, with statistically significant differences (*P*=0.01). The mean OHIP-14 parameters in the two groups did not reveal any differences in relation to the subjects’ age, gender and educational status.  Table 5 shows that in the comparison of the fields of the quality of life under study between the two groups. Based on the Linear regression model, in patients with maxillofacial trauma compared to dental surgery group; a significant difference was observed in the all of domains(*P*<0.05).

**Table 5:**
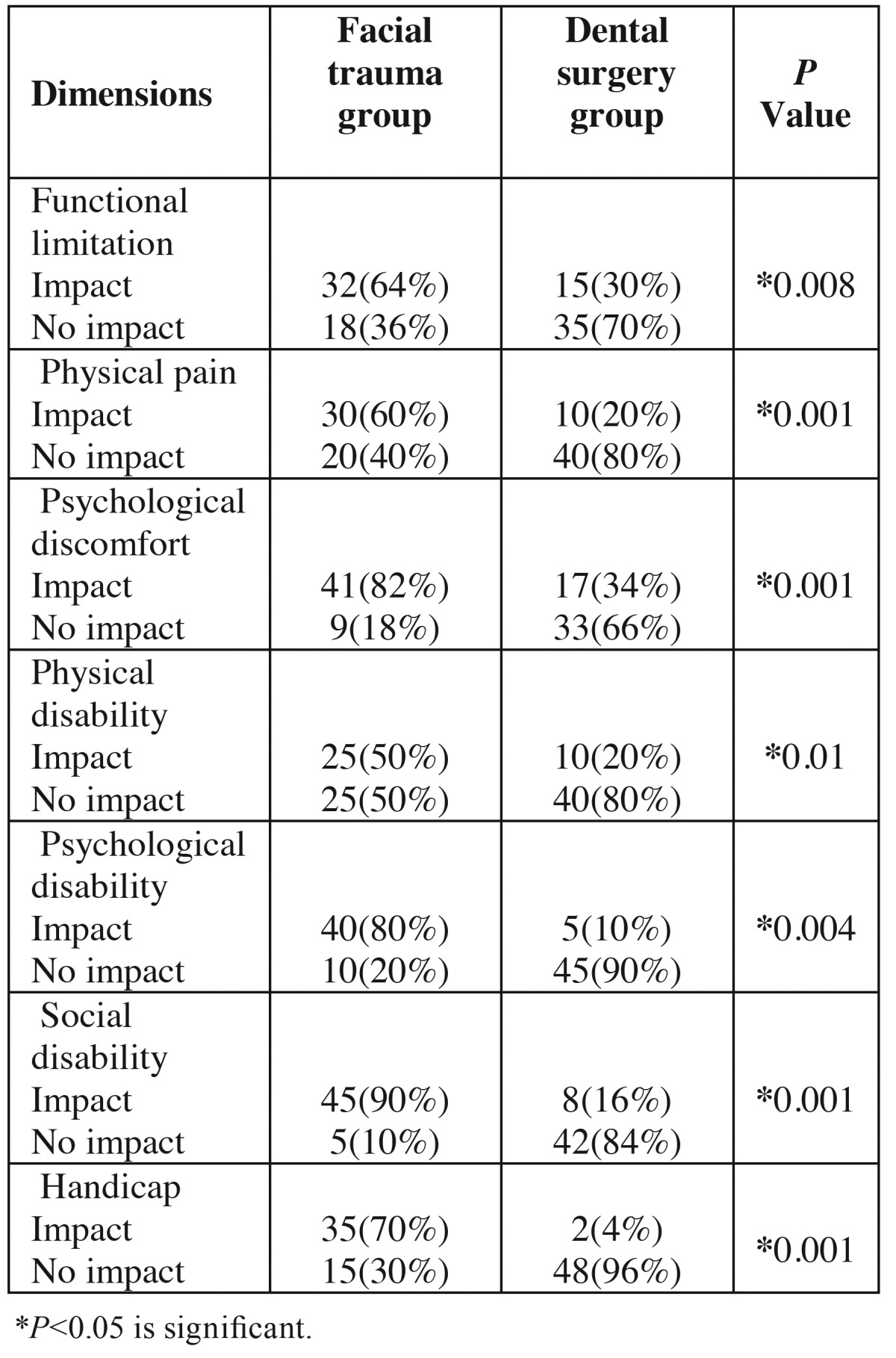
Frequency Distribution of Reported Impacts on the 7 Domains of the Oral Health Impact Profile Measure (OHIP-14) in Facial trauma and Dental surgery groups.

## Discussion

Although maxillofacial fractures are one of the most common injuries in treatment centers, the emotional and behavioral disorders that remain untreated in these patients, including depression, antisocial behaviors, lack of occupational achievements, drug abuse and risky behaviors that increase the risk of being injured again, do not attract attention (14-16). In addition, despite significant advances in medicine and dentistry, treatment and surgery of these injuries is still a challenge. Traumas resulting from the specific functions in this area might be the reason for psychological disorders in these patients. In addition, such traumas might result in facial deformities or pave the way for chronic diseases and the psycho-social effects of these traumas might be long-lasting (17).

Studies by Freedman *et al.* (18) and Harvey and Bryant (19) have shown the incidence of depression, initial development of depression and anxiety symptoms and signs in these patients during the first two years after trauma.

The majority of studies on patients with permanent facials injures have focused on the evaluation of surgical interventions and the results of treatments rendered. Although it has been shown that changes in the facial appearance due to traumas might give rise to adaptation problems, relatively few studies have evaluated the psychological status after maxillofacial traumas (6).

In the present study, depression, anxiety and quality of life were evaluated in 2 groups of patients with maxillofacial traumas and patients referring for surgeries on their teeth (including tooth extraction). The results of the present study showed higher levels of anxiety and depression in patients with maxillofacial traumas compared to the other group, and the differences were significant.

Previous studies have shown that clinicians do not pay much attention to the psycho-social injures symptoms in trauma (7,2). Recent studies on observations made by surgeons treating maxillofacial injuries have shown that less than half of the clinicians believe that adequate attention has been paid to the psychological well-being of patients in hospitals (12).

The present study showed that 24% of patients with maxillofacial traumas exhibited some degrees of depression and 32.5% had some degrees of anxiety. A study by Islam *et al.* (6) showed that 20% of patients exhibited some degrees of anxiety and depression disorders.

Hull *et al.* (4,5) evaluated the psychological outcomes of maxillofacial traumas in 39 patients and reported that 13% of patients exhibited mild to moderate depression during the initial interview. A review of medical research studies showed a prevalence rate of 11.8-32% in these patients (2,9,8,20,21).

Evaluation of maxillofacial traumas in Australia showed that 11.5% and 15% of patients had some degrees of depression and anxiety, respectively, with the anxiety levels of 11.5% and 15% during the initial phase after maxillofacial traumas in studies by Hull *et al.* (4,5) and Ukpong *et al.* (22), respectively. The differences in the prevalence rates of anxiety and depression in different studies might be attributed to the use of different tools to evaluate these problems and differences in different study populations in relation to age and cultural and social factors. For example, Hull *et al.* (4,5) used clinical interviews in their study, while in the present study a psychometric questionnaire was used. Another explanation might be a relatively higher percentage of subjects in the present study with psychological problems before infliction of trauma.

A literature review in medical publish paper shows this important fact that in a great number of studies about psychological effects of traumatic injuries high emphasis is made on delayed psychological reactions, most notably post-traumatic anxiety disorder. As an interestingly results, one can mention, to this fact that there would appear to be considerable variations in the PTSD rate published in the literature with many research work reporting prevalence of ranging from 1.9% to 33% 12 months after trauma (20,21). From all adults with maxillofacial injuries, between 20% and 30% of have been reported to have symptoms of PTSD (2,9,8).

The results of the present study showed that the incidence rate of depression had significant relationships with gender (higher in females compared to males), occupation (higher in the unemployed compared to the employed), a history of taking antidepressant medications and a history of psychological problems. Clinical and epidemiological studies have shown a higher rate of depression in women compared to men (22,6,9,14).

Studies on facial traumas have shown that the severity of anxiety has a direct relationship with the extent of trauma (23,6). Other studies have shown that patients’ perception of changes in their facial appearance is an important factor in developing anxiety and depression (6). Female gender, permanent injuries, aging, chronic pain due to the trauma and a history of psychological problems might increase the odds of psychological outcomes subsequent to maxillofacial traumas (2,6). The results of the present study are consistent with those of studies by Shepherd *et al.* and Islam *et al.* (7,6) who evaluated the psychological outcomes of patients with maxillofacial fractures. They reported significant differences in depression and anxiety between patients with maxillofacial traumas and the healthy controls. However, Bisson *et al.* (2) did not report any significant relationship between such traumas and depression and anxiety.

It was revealed in some facial trauma studies that the degree of anxiety is directly proportional to the magnitude of injury and the scar it leaves (23,6). It is reported in several research works that in the early period after maxillofacial trauma, the anxiety rate is in the range of 11.5% to 15% (3,6,5). While the rates of depression after facial trauma have been reported 8-13% (5,6).

The results of the present study showed an eight-fold increase in the risk of depression (OR=8.12) and 

a two-fold increase in the risk of anxiety (OR=2.25) in patients with maxillofacial traumas compared to the control group, consistent with studies in Italy.

The results of the present study showed that patients with mandibular traumas had higher levels of anxiety compared to those with maxillary traumas, consistent with other studies (1,7-9).

The present study showed poorer quality of life in patients with maxillofacial traumas compared to the other group, with significant differences, consistent with the results of a study by others (24-26).

This study showed that it is very important to provide psychiatric support for all the patients with maxillofacial traumas. Clinicians should emphasize this important consideration and explain it to the patients’ relatives in emergency departments and care units.

## Conclusions

The results of the present study showed depression and anxiety disorders in patients with maxillofacial trauma. The results showed a higher rate of anxiety and anxiety in patients with maxillofacial traumas compared to the control group.
